# A Comparative Study: Has MRI-guided Fusion Prostate Biopsy Changed the Prostate-specific Antigen Gray-zone Range?

**DOI:** 10.7759/cureus.6329

**Published:** 2019-12-08

**Authors:** Gökhan Sönmez, Şevket T Tombul, Türev Demirtaş, Figen Öztürk, Abdullah Demirtaş

**Affiliations:** 1 Urology, Kayseri City Hospital, Kayseri, TUR; 2 Urology, Erciyes University, Kayseri, TUR; 3 History of Medicine and Ethics, Erciyes University, Kayseri, TUR; 4 Pathology, Erciyes University, Kayseri, TUR

**Keywords:** cut-off, fusion, psa, prostate, gray-zone

## Abstract

Objective

The gray-zone prostate-specific antigen (PSA) range is accepted to be 4-10 ng/ml and is considered to vary according to age. We aimed to investigate whether fusion prostate biopsy (FPB), which has been reported to have relatively higher cancer detection rates, has an effect on gray-zone PSA cut-off value.

Material and methods

This retrospective study included patients that underwent standard prostate biopsy (SPB) or multiparametric magnetic resonance imaging (MpMRI)-guided FPB (SPB+ targeted biopsy). All the patients included in the study were detected with a Prostate Imaging Reporting and Data System (PI-RADS) ≥3 lesion on MpMRI (the FPB group only). The demographics, clinical characteristics, and histopathological diagnoses were recorded for each patient.

Results

A total of 1,628 patients comprising 1,208 patients in the SPB group and 420 patients in the FPB group were included in the study. The mean PSA level was 9.75±6.68 ng/ml in the FBP group and 10.46±6.46 ng/ml in the SPB group (p=0.053). Prostate cancer (PCa) detection rate was significantly higher in the FPB group as compared to the SPB group (42.4% vs. 36.4%). The PSA cut-off value for PCa was 9.75 ng/ml (sensitivity and specificity, 81%) in the SPB group and was 7.55 ng/ml (sensitivity and specificity, 81% and 84%, respectively) in the FPB group. In the FPB group, the cancer detection rate among the patients with a PSA level of 7.55-10.00 ng/ml was 56.1%.

Conclusion

The results indicated that the introduction of FPB into clinical practice, which has relatively higher cancer detection rates, has further lowered the upper limit for gray-zone PSA.

## Introduction

Prostate cancer (PCa) is the second-most common cancer in men worldwide [1]. Prostate-specific antigen (PSA) is the most commonly preferred serum marker in patients with suspicious PCa and plays a key role in prostate biopsy decision-making. However, PSA is an organ-specific but not a cancer-specific marker and may be elevated in nonmalignant conditions such as benign prostate hyperplasia and prostatitis [2-3].

There is no consensus in the literature regarding the normal PSA range. However, the upper limit for PSA is often accepted to be 2.5 or 4.0 ng/ml, and it is considered to vary according to age [4-5]. The same situation is valid for the definition of gray-zone PSA. Although some studies suggest that the gray-zone PSA range may show regional and ethnic variation, some other studies have proposed an upper limit of 10 ng/ml for gray-zone PSA [6-9].

Multiparametric magnetic resonance imaging (MpMRI)-guided fusion prostate biopsy (FPB) is a recently developed biopsy technique. This technique has been shown to provide effective outcomes. FPB can be performed in the form of cognitive, fusion, or in-bore biopsy using ultrasound fusion imaging with a computer program [10]. Numerous studies have indicated that FBP provides higher PCa detection rates as compared to 10-12 core standard transrectal prostate biopsy (SPB), particularly at low PSA levels [11-13].

The present study aimed to investigate whether FPB, which has relatively higher cancer detection rates, has an effect on gray-zone PSA cut-off value by comparing the PSA cut-off values and gray-zone PSA ranges for SPB and FPB.

## Materials and methods

Patients

The retrospective study included patients aged 45-75 years that underwent either 10-12 core SPB between January 2010 and December 2016 or mpMRI-guided FPB (12 core SPB + targeted biopsy (TB)) between January 2017 and April 2019. Inclusion criteria were elevated PSA levels and/or the presence of suspected rectal examination-suspicious radiological imaging. Patients with a Prostate Imaging Reporting and Data System (PI-RADS) <3 lesion, PSA >50 ng/ml, incomplete medical records, a histopathological diagnosis of atypical small acinar proliferation (ASAP), or high-grade prostatic intraepithelial neoplasia (HGPIN), and patients that underwent SPB with fewer than 10 cores or more than 12 cores were excluded from the study. Demographic and clinical characteristics, including age, body mass index (BMI), serum PSA level, total prostate volume, number of biopsy cores, and histopathological diagnosis, were recorded for each patient.

Multiparametric MRI and Prostate Imaging-Reporting and Data System

Prior to FPB, each patient underwent prostate mpMRI without endorectal coil using a Siemens Magnetom 1.5 Tesla MRI device (Siemens Medical Solutions, Malvern, USA). Suspicious lesions detected on contrast-enhanced T1-, T2-, and diffusion-weighted sequences were recorded based on PI-RADS version 2 [14]. In patients with more than one lesion and multiple PI-RADS scores, only the highest PI-RADS score was considered.

Pre-biopsy

Prior to biopsy, each patient gave a sterile urine culture. The ongoing anticoagulant and antiaggregant treatments of the patients were stopped. Twenty-four hours before the procedure, three oral doses of 750 mg of ciprofloxacin were administered at a 12-h interval. No bowel preparation was performed in any patient prior to the procedure. Biopsy procedures were performed under local or sedation anesthesia in the outpatient clinics according to patient preference.

Standard prostate biopsy

SPB was performed under transrectal ultrasonography (TRUS) guidance with 10-12 cores. Local anesthetics were administered, followed by the injection of intrarectal lignocaine gel + prilocaine combination. Following the induction of periprostatic nerve blockage, total prostate volume was measured using the following formula: height (H) x width (W) x length (L) x 0.523.

Fusion prostate biopsy

FPB was performed by using an ultrasonography (US) system with rigid fusion software and an endorectal single-angle probe. Local anesthetics were administered, followed by the injection of intrarectal lignocaine gel + prilocaine combination. The presence of prominent lesions was determined by a sonographic examination of the prostate tissue. Total prostate volume was measured using the following formula: H x W x L x 0.523. FPB was performed with 12 cores and then the mpMRI images were transferred to the US system. Segmentation (matching) of sonographic images with MR images was performed and the lesions detected on mpMRI were marked. For each lesion marked on mpMRI, FPB (SPB + TB) was performed using two to five cores for each lesion. All the data transfers and markings throughout the procedure were performed by two urologists experienced and trained in transrectal prostate ultrasonography and biopsy.

Histopathological examination

The specimens were sent for a histopathological examination in previously labeled containers. The cancer detection rate per core was calculated in accordance with the International Society of Urological Pathology (ISUP) grading system, which is based on primary and secondary Gleason scores [15]. Clinically significant prostate cancer (sPCa) was considered as biopsy Gleason score ≥3+4 or maximum cancer core length ≥5 mm.

Statistical analysis

Data were analyzed using Statistical Package for Social Sciences (SPSS) for Windows version 22.0 (Armonk, NY: IBM Corp.). Normal distribution of data was assessed with the Shapiro-Wilk test and histogram plots. Based on the distribution pattern, continuous variables were presented as mean ± standard deviation (SD) or as median (1-3rd quartile). Categorical variables were expressed as percentages (%). In independent groups, normally distributed continuous variables were compared using independent-samples t-test and non-normally distributed continuous variables were compared using the Mann-Whitney U test. The cut-off value was considered as the point with the maximum sensitivity and specificity and was determined using a Receiver Operating Characteristic (ROC) curve analysis. A p-value of <0.05 was considered significant.

## Results

A total of 1,628 patients comprising 1,208 (74.2%) patients in the SPB group and 420 (25.8%) patients in the FPB group were included in the study. The mean PSA level was 10.28 ± 6.52 ng/ml. Of all the patients, 38% of them were detected with sPCa, among whom ISUP grade 1 was the most common histopathological diagnosis (17.8%). Table 1 presents some demographic and clinical characteristics of the patients.

**Table 1:** Some demographic and clinical data of all patients PSA: Prostate-specific antigen, ISUP: International society of urological pathology, SPB: Standard prostate biopsy, FPB: Fusion prostate biopsy, TB: Target biopsy

Variable	Value
Age (year)	66.0 (61.0-71.0)
Body Mass Index (BMI) (kg/m^2^)	25.85 (23.50-28.50)
PSA (ng/ml)	10.28 (+/- 6.52)
Total prostate volume (mm^3^)	59.00 (41.00-84.00)
Overall cancer detection rate (%)	41.8
Clinically significant cancer detection rate (%)	38.0
ISUP score (%): 1; 2; 3; 4; 5	46.8; 16.1; 12.1; 13.2; 11.8
Biopsy Type (n): SPB/ FPB (SPB + TB)	1,208/ 420

The two groups were similar with regard to PSA level and median age (p=0.053 and p=0.085, respectively). Overall PCa and sPCa detection rates were significantly higher in the FPB group as compared to the SPB group (Table 2).

**Table 2:** Comparison of data from the standard and fusion prostate biopsy groups PSA: Prostate-specific antigen, SPB: Standard prostate biopsy, FPB: Fusion prostate biopsy

Parameter	SPB (n=1,208)	FBP (n=420)	p
Age (year)	66.0 (61.0-71.0)	65.0 (60.0-70.0)	0.085
PSA (ng/ml)	10.46 (+/- 6.46)	9.75 (+/- 6.68)	0.053
Total prostate volume (mm^3^)	59.00 (41.00-85.00)	57.50 (42.00-79.00)	0.561
Overall cancer detection rate (%)	40.4	46.0	0.047
Clinically significant cancer detection rate (%)	36.4	42.4	0.030
Number of cores (n)	12.0 (11.0-12.0)	15.0 (15.0-17.0)	<0.001

**Figure 1:**
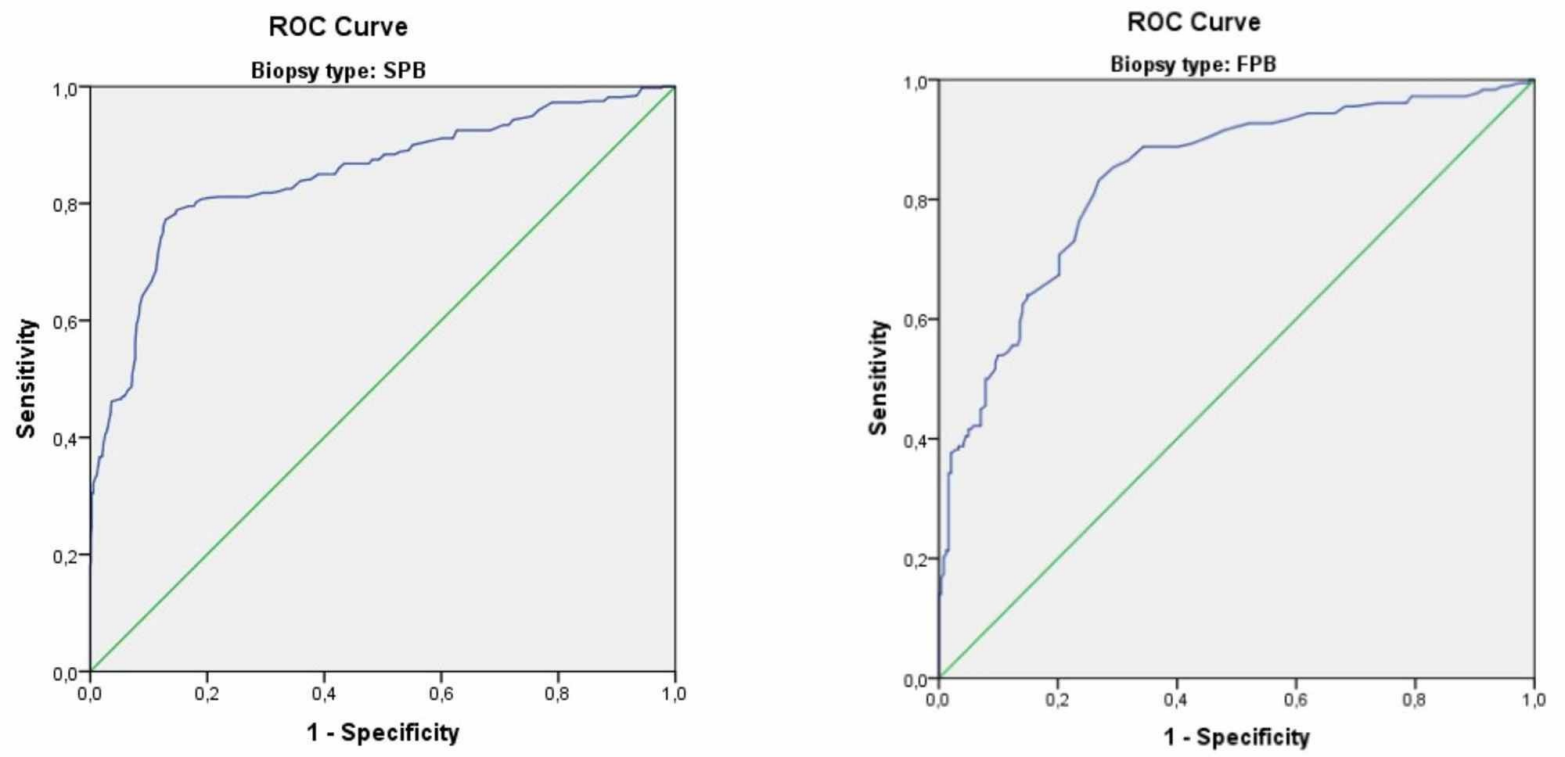
The Receiver Operating Characteristic (ROC) curve analysis for prostate-specific antigen in the fusion prostate biopsy and standard prostate biopsy groups

In the ROC curve analysis, the area under the ROC curve (AUC) for PSA was 0.837 in the FPB group and 0.849 in the SPB group and a strong correlation was found between PCa and PSA in both groups (Figure 1). The PSA cut-off value for PCa was 9.75 ng/ml with a sensitivity and specificity of 81% in the SPB group and was 7.55 ng/ml with a sensitivity and specificity of 81% and 84%, respectively, in the FPB group (Table 3). In the FPB group, the cancer detection rate among the patients with a PSA level of 7.55-10.00 ng/ml was 56.1% (Table 4).

**Table 3:** Relationship between the standard prostate biopsy and fusion prostate biopsy groups with PSA SPB: Standard prostate biopsy, FPB: Fusion prostate biopsy, AUC: Area under curve, SE: Standard error, CI: Confidence interval, PSA: Prostate-specific antigen

Biopsy type	AUC	SE	p	%95 CI	Sensitivity, %	Specificity, %	PSA cut-off value (ng/ml)
SPB	0.849	0.012	<0.001	[0.824, 0.873]	81	81	9.75
FPB	0.837	0.020	<0.001	[0.798, 0.876]	81	84	7.55

**Table 4:** FPB and SPB cancer detection rates according to PSA intervals Different superscripts given in the same line indicate a statistically significant difference. FBP: Fusion prostate biopsy, SPB: Standard prostate biopsy, PSA: Prostate specific antigen

Biopsy type	PSA range-cancer detection rates			
	0-7.5 ng/ml	7.6-10 ng/ml	>10 ng/ml	P-value
SPB	%12.6 (58/460)^a^	%11.9 (32/269)^a^	%73.1 (350/479)^b^	Pab<0.001
FPB	%15.9 (34/213)^x^	%56.1 (55/98)^y^	%81.6 (89/109)^z^	Pxy, Pyz<0.001

## Discussion

In the SPB group, the PSA cut-off value for PCa was 9.75 ng/ml, which appears consistent with the commonly accepted gray-zone PSA level (10 ng/ml). In the FPB group, however, the PSA cut-off value for PCa was 7.55 ng/ml, which supports the hypothesis that the upper limit for gray-zone PSA has been further lowered with the introduction of FPB into clinical practice. On the other hand, the cancer detection rate in the FPB group among the patients with a PSA of 7.55-10.00 ng/ml was 56.1%, which implicates that this reference range cannot be accepted as the gray-zone range.

In Europe, prostate cancer is the second most common cancer in men and age-adjusted prostate cancer incidence rate was 35 cases per 100,000 in Turkey [1,16]. The most commonly used marker in the diagnosis of prostate cancer is PSA [2]. The upper limit for gray-zone PSA has been determined as 10 ng/ml in numerous studies [6-8,15,17-18]. Some other studies, though few in number, suggest that the upper limit for gray-zone PSA may show ethnic variation and propose that this limit is likely to be higher in countries where PCa is less common, particularly in Far Eastern countries [9,19]. Zhao et al. [6] determined the gray-zone PSA range as 4-10 ng/ml. Based on this range, the authors detected PCa in 17% of the patients that underwent SPB and in 50% of the patients with a gray-zone PSA level of 10-50 ng/ml. The authors suggested that these rates confirmed the accuracy of the PSA range determined in the study. In our study, sPCa was detected in 12.3% of the patients in the SPB group who had a PSA level of 0-10 ng/ml (Table 4). The difference between our study and the study by Zhao et al. could be attributed to the inclusion of patients with a PSA level of 0-4 ng/ml in that study. Nevertheless, our study was consistent with the study by Zhao et al. in that the upper limits for gray-zone PSA level determined for SPB in both studies were consistent with the commonly accepted upper limit (10 ng/ml). In a recent study, Creed et al. [8] evaluated a total of 126 patients with PSA <10 ng/ml who underwent FPB and reported an sPCa detection rate of 24.6%. The same study, however, performed no gray-zone calculation for any patient in the FPB group and the study, unlike our study, included only patients with a history of negative prostate biopsy. In our study, the PCa detection rate was 28.6% in the patients with a PSA <10 ng/ml who underwent FPB. Another study rejected the commonly accepted upper limit for gray-zone PSA (10 ng/ml) and found similar PCa detection rates for patients with a PSA of 4-10 ng/ml and 10-20 ng/ml (20.5% vs. 21.6%; p=0.854) [9]. The authors contended that this similarity could be associated with ethnic variation and to the lower incidence of PCa in Chinese men. In a study conducted in 2014, Sarıkaya et al. [18] found a similar PCa detection rate (12.4%) to that of our study in patients with a PSA level of 4-10 ng/ml who underwent SPB.

FPB is known to detect more prostate cancer than SPB at low PSA levels [10-13]. According to our results, in patients with PSA levels below 7.5 ng/ml, there were small differences between the cancer detection rates of biopsy methods (12.6% vs 15.9%). In contrast, FPB was found to be superior, especially at PSA levels of 7.5-10 ng/ml (11.9% vs 56.1%). Therefore, our results show that FPB should be preferred, especially in the range of PSA 7.5-10 ng/ml.

Boasen et al. [12], in their 2014 study, detected sPCa in 43% of the patients that underwent FPB (SPB + TB) and in 36% of the patients that underwent SPB alone. Another study reported the overall PCa and sPCa detection rates as 46.1% and 33.5%, respectively. The authors noted that the sPCa detection rate in the FPB (SPB + TB) group was 45% and suggested that FPB is superior to SPB [19]. Similarly, Fourcade et al. [20] reported that the best biopsy method was combined biopsy. In our study, PCa and sPCa detection rates were 42.4% and 36.4%, respectively, which were significantly higher in the FPB group as compared to the SPB group (p=0.47 and p=0.30, respectively) and were consistent with the literature.

In our patients, ISUP grade 1 was the most common histopathological diagnosis (46.8%). Similarly, a 2013 study that evaluated 582 patients also reported that ISUP grade 1 was the most common histopathological diagnosis in the patients [21]. A more recent study, however, found ISUP grade 2 as the most common histopathological diagnosis in the patients [12]. The patients included in that study had higher PSA levels compared to those of our patients (12.80 vs. 10.28 ng/ml) and the study included only patients that underwent FPB. Taken together, these findings implicate that ISUP grade 1 and 2 tumors are likely to be detected at low PSA levels and our findings are consistent with the literature.

Our study has several key limitations. First, the study had a retrospective design. Second, a large patient series is needed for the determination of a novel and definitive cut-off value for gray-zone PSA. Therefore, the relatively small number of patients in the FPB group is an important limitation of our study. Third, the presence of a well-established cancer marker (i.e. PI-RADS ≥3 lesion) in our patients that underwent FPB may lead to partial bias. This could be accepted as a limitation of our study; however, it should be recognized that FPB and the presence of a PI-RADS ≥3 lesions are highly integrated with each other. Fourth, patients that underwent FPB and had a histopathological diagnosis of ASAP and HGPIN were excluded from the study and no clear information could be obtained regarding their treatment outcomes due to their short follow-up period, which might have led to the acquisition of results that could be slightly different from those of daily practice. Finally, an age-based PSA cut-off value could not be calculated as there were very few patients in some age groups.

## Conclusions

The results indicated that the introduction of FPB into clinical practice, which has relatively higher cancer detection rates, has further lowered the upper limit for gray-zone PSA. Accordingly, we suggest that the upper limit for gray-zone PSA should be revised to 7.5 ng/ml and the clinics administering FPB should be more courageous in making biopsy decisions in patients with a PSA level of >7.5 ng/ml.
